# Effect of preoperative chemotherapy on postoperative liver regeneration following hepatic resection as estimated by liver volume

**DOI:** 10.1186/1477-7819-11-65

**Published:** 2013-03-13

**Authors:** Daiki Takeda, Hiroyuki Nitta, Takeshi Takahara, Yasushi Hasegawa, Naoko Itou, Go Wakabayashi

**Affiliations:** 1 Department of Surgery, Iwate Medical University, 19-1 Uchimaru, Morioka, 020-8505, Japan

## Abstract

**Background:**

In order to analyze postoperative liver regeneration following hepatic resection after chemotherapy, we retrospectively investigated the differences in liver regeneration by comparing changes of residual liver volume in three groups: a living liver donor group and two groups of patients with colorectal liver metastases who did and did not undergo preoperative chemotherapy.

**Methods:**

This study included 32 patients who had at least segmental anatomical hepatic resection. Residual liver volume, early postoperative liver volume, and late postoperative liver volume were calculated to study the changes over time. From the histopathological analysis of chemotherapy-induced liver disorders, the effect on liver regeneration according to the histopathology of noncancerous liver tissue was also compared between the two colorectal cancer groups using Kleiner’s score for steatohepatitis grading {Hepatology, **41**(6):1313–1321, 2005} and sinusoidal obstruction syndrome (SOS) grading for sinusoidal obstructions {Ann Oncol, **15**(3):460–466, 2004}.

**Results:**

Assuming a preoperative liver volume of 100%, mean late postoperative liver volumes in the three groups (the living liver donor group and the colorectal cancer groups with or without chemotherapy) were 91.1%, 80.8%, and 81.3%, respectively, with about the same rate of liver regeneration among the three groups. Histopathological analysis revealed no correlation between either the Kleiner’s scores or the SOS grading and liver regeneration.

**Conclusions:**

As estimated by liver volume, the level of liver regeneration was the same in normal livers, tumor-bearing livers, and post-chemotherapy tumor-bearing livers. Liver regeneration was not adversely affected by the extent to which steatosis or sinusoidal dilatation was induced in noncancerous tissue by chemotherapy in patients scheduled for surgery.

## Background

Colorectal cancer remains a major cause of cancer deaths throughout the world. Survival rates are strongly related to how extensively distant metastases are present. The liver is often the site of distant metastatic involvement. Approximately 25% of patients reportedly have liver metastases when diagnosed with colorectal cancer, and 25–35% of patients who have undergone resection for primary lesions show liver metastases [1].

Hepatic resection is potentially curative in colorectal cancer patients with liver metastases. In resectable cases, a 5-year survival rate of more than 50% can be anticipated [2]. However, only 15–20% of patients are considered resectable. This is because the liver metastases have spread to both lobes or because adequate residual liver volume cannot be ensured. Meanwhile, chemotherapy is an option in other patients who are considered unresectable. Some of these patients become resectable after conversion chemotherapy, and the importance of multimodal therapy that combines conversion chemotherapy and surgical resection in such cases has been reported [3,4].

Intra- and extrahepatic recurrences are often detected after hepatic resection. The usefulness of neoadjuvant chemotherapy in advance of hepatic resection is being increasingly claimed, but its appropriateness is controversial. Some advantages of neoadjuvant chemotherapy are that the shrinkage of liver metastases enables R0 resection [5] and that such neoadjuvant chemotherapy prior to hepatic resection can eradicate micro-lesions and prolong progression-free survival [6,7]. Conversely, some disadvantages are that chemotherapy causes the potential for more operative complications [8], greater hepatic impairments [9-11], and procedural difficulties in the event of complete response (CR) on imaging [12]. And it has not yet been decided how long the patients had undergone neoadjuvant chemotherapy. Preoperative chemotherapy may result in chemotherapy-related liver disorders, particularly in colorectal cancer patients with liver metastases [13]. Steatohepatitis and sinusoid obstruction are considered common liver disorders. Steatohepatitis is said to be strongly correlated to irinotecan, while sinusoidal obstruction is said to be strongly correlated to oxaliplatin. These disorders may also result in more perioperative complications [10-14].

The indocyanine green retention rate at 15 min (ICGR15) is one of some useful measures of preoperative hepatic functional reserve prior to hepatic resection, and the extent of hepatic resection in hepatitis and obstructive jaundice has been amply reported to date [15-17]. Few reports have described methods for rigorously assessing the hepatic reserve after preoperative chemotherapy in colon cancer patients with liver metastases.

The key point of this study was that the level of liver regeneration in tumor-bearing livers and post-chemotherapy tumor-bearing livers was revealed to be consistent with that of normal livers by liver volume estimation. Another important point was that we monitored the regeneration rate of the liver from the living liver donor as a control.

## Methods

This study included 32 patients who had anatomical hepatic resection. We compared three groups, composed of 17 colorectal cancer patients with liver metastases who had undergone at least segmental anatomical hepatic resection (8 patients with and 9 patients without preoperative chemotherapy) and 15 patients in a living liver donor transplantation group in our department since January 2008. The preoperative chemotherapy regimen was oxaliplatin-based in six subjects, irinotecan-based in one subject, and oxaliplatin- and irinotecan-based in one subject. Modern regimens include biologic agents or “antibodies” such as bevacizumab or cetuximab. The preoperative washout was a minimum of 4 weeks (mean, 11.6 weeks) (Table 1). In addition, in colon cancer patients with multiple liver metastases, at least 30% residual liver volume must be ensured, and hepatic resection cannot be performed any sooner than 4 weeks after chemotherapy.

**Table 1:** Preoperative chemotherapy regimen, duration (number of course), and time before hepatectomy (preoperative washout)

**Regimen**	**Case**	**Number of case**	**Preoperative washout (weeks)**
**FOLFOX4**	1 (12.5%)	14	4
**mFOLFOX6 + BEV**	3 (37.5)	8.3	14
MFOLFOX6 + CET**	1 (12.5%)	4	4
**MFOLFOX6→sLV + 5FLU**	1 (12.5%)	9 → 3	24
**FOLFIRI**	1 (12.5%)	51	4
**mFOLFOX6→FOLFIRI**	1 (12.5%)	3 → 6	8

BEV^*^: bevacizumab, CET^**^: cetuximab.

This study is the retrospective clinical one planned by the Department of Surgery, Iwate Medical University School of Medicine. Patient data were retrospectively gained from our database for colorectal liver metastases patients and living liver donors.

### Liver volume analysis

Preoperative liver volume, residual liver volume, early postoperative liver volume, and late postoperative liver volume in each group were calculated and analyzed. The early postoperative period was defined as about 1 month postoperatively (median 1.1, range 1.0–1.1), and the late postoperative period was defined as about 6 months postoperatively (median 6.2, range 5.8–6.3). Liver volumes were measured using a SYNAPSE VINCENT volume analyzer (Fujifilm Co., Ltd., Japan) to measure the volume of reconstructed three-dimensional liver images based on CT performed at each analysis time point, and changes over time were compared among the three groups.

To exclude the effects of the preoperative tumor loads, we have withdrawn the volume of the liver metastases from the total liver volume to calculate a “functional liver volume.” The liver regeneration rate versus the “functional liver volume” was also calculated in tumor-bearing livers (*n* = 9) and post-chemotherapy tumor-bearing livers (*n* = 8) at each analysis time point.

### Histopathological analysis

Chemotherapy-induced hepatic impairment was histopathologically analyzed between groups of subjects who had and had not undergone preoperative chemotherapy. All histopathological assessments were undertaken by the same pathologist. Steatohepatitis was assessed by grading the severity of each parameter (steatosis, fibrosis, inflammation, liver cell injury, and other findings) using Kleiner’s score to calculate the total score (Table 2) [18]. Sinusoidal obstructions were assessed using the three-stage grading for sinusoidal obstruction syndrome (SOS), where centrilobular sinusoidal obstruction of no more than one-third was considered “mild,” and sinusoidal obstructions of two-thirds or more of both lobes were considered “severe,” with “moderate” in between the two [14].

**Table 2:** **Grading the severity of each parameter using Kleiner’s score**[1]

			**Score**
Steatosis	Grade	<5%	0
		5–33%	1
		33–66%	2
		>66%	3
	Location	Zone 3	0
		Zone 1	1
		Azonal	2
		Panacinar	3
	Microvesicular steatosis	Not present	0
		Presented	1
Fibrosis stage		None	0
		Perisinusoidal or periportal	1
		Perisinusoidal and periportal/periportal	2
		Bridging fibrosis	3
		Cirrhosis	4
Inflammation	Lobular inflammation	No foci	0
		<2 foci per 200× field	1
		2–4 foci per 200× field	2
		>4 foci per 200× field	3
Liver cell injury	Ballooning	None	0
		Few balloon cells	1
		Many cells/ prominent ballooning	2
	Acidophil bodies	None to rare	0
		Many	1
	Pigmented macrophages	None to rare	0
		Many	1
Other findings	Mallory's hyaline	None to rare	0
		Many	1
	Glycogenated nuclei	None to rare	0
		Many	1

### Operation procedure

Hepatectomy was performed anatomically, using the hepatic vein as the landmark for dissection, where the surgeon made use of CUSA and the first assistant made use of a coagulation hemostatic device, with the intention of achieving the least possible congestion in the residual liver.

### Statistics

Continuous variables are expressed as mean ± standard error of the mean. Differences among the three groups were analyzed by the Kruskal-Wallis test, as appropriate. Statistical significance was accepted at *p* < 0.05. Data analysis was performed with Stat-View 5.0 (SAS Institute, Cary, NC).

## Results

The mean ages in the living liver donor group, nonchemotherapy group, and chemotherapy group were 36.6 years (range, 20–57 years), 68.0 years (range, 53–78 years), and 59.6 years (range, 42–66 years), respectively, and the male:female ratios were 8:7, 8:1, and 6:2, respectively (Table 3).

**Table 3:** Profiles of the enrolled patients

	**Living liver donor (*n*** **= 15)**	**Without chemotherapy (*n*** **= 9)**	**Without chemotherapy (*n*** **= 8)**
Age (mean ± SE*)	36.6 ± 3.1	68.0 ± 2.3	59.6 ± 3.0
**Sex, M/F**	8/7	8/1	6/2
**Operative method**			
**(hepatectomy)**			
**Major**	15	6	6
**Minor**		3	2
**Operative time (min)**	381.6 ± 9.5	238.2 ± 28.8	305.9 ± 25.4
**(mean ± SE)**	294.2 ± 36.7	450.9 ± 102.6	595.0 ± 249.7
**Blood loss (cc) (mean ± SE)**	8.4 ± 0.6	10.1 ± 1.3	27.6 ± 12.3
**(mean ± SE)**	1(Clavia IIIa)/0	2(C lavian IIa)/0	1(Clavian IIIa)/0
**Morbidity/mortality**			

Continuous variables are expressed as mean ± SE* (standard error of the mean).

**Figure 1:**
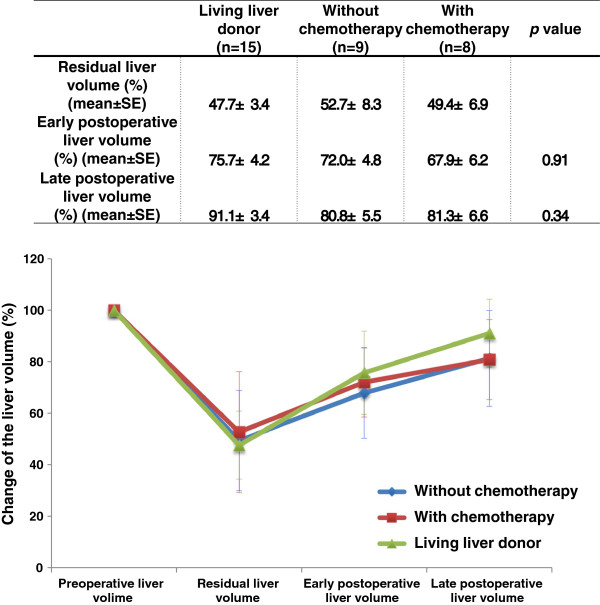
The changes over time in liver volume among the three groups.

The changes over time in liver volume in each group were graphed (Figure 1). Differences were expressed as the rate of increase assuming a preoperative liver volume of 100%. In the living liver donor group, the mean postoperative residual liver volume was 47.7% of the total preoperative liver volume, increasing about 28% to 75.7% in the early postoperative period, with liver regeneration up to 91.1% by the late postoperative period. In the nonchemotherapy group, the mean postoperative residual liver volume was 52.7%, increasing to 72.0% in the early postoperative period, revealing approximately 20% liver regeneration. Liver regeneration up to 80.8% was ultimately found by the late postoperative period. In the chemotherapy group, the mean residual liver volume was about 49.4% of the total preoperative liver volume, with an approximately 18% increase in liver volume, to 67.9% in the early postoperative period. Liver regeneration up to about 81.3% was found by the late postoperative period. The mean liver volume in the three groups is shown in a graph (Figure 1). The results showed that the changes over time in liver volume were similar in all three groups. The rate of liver regeneration was about the same in the three groups. In tumor-bearing liver group (*n* = 9), the mean postoperative residual liver volume was 54.4% of the “functional liver volume,” increasing to 73.2% in the early postoperative period, with liver regeneration up to 85.5% by the late postoperative period. In post-chemotherapy tumor-bearing liver group (*n* = 9), the mean postoperative residual liver volume was 56.9% of the “functional liver volume,” increasing to 80.4% in the early period and 97.7% in the late period. The results for liver regeneration versus the “functional liver volume” in the two groups are shown as almost even.

The liver regeneration rate versus the hepatic resection volume was also calculated. In the living liver donor group, a mean liver regeneration of 54.4% was found in the early postoperative period, and 28.2% regeneration was found in the late postoperative period. In the nonchemotherapy group, a mean liver regeneration of 36.3% was found in the early postoperative period, and a mean regeneration of 30.4% was found in the late postoperative period. In the chemotherapy group, a mean liver regeneration of 57.5% was found in the early postoperative period, and 36.5% regeneration was found in the late postoperative period. The results for liver regeneration versus hepatic resection volume in the three groups are shown.

**Figure 2:**
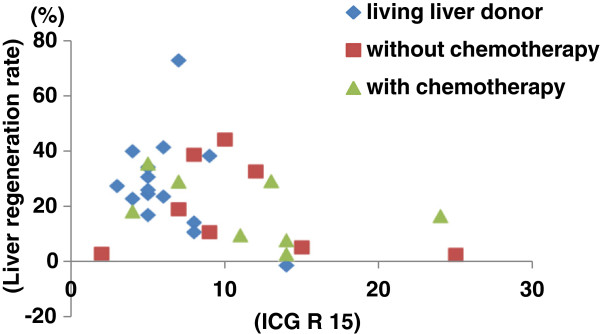
The relationship between ICG R15 and liver regeneration.

The relationship between ICG R15 and liver regeneration is shown (Figure 2). The liver regeneration rate (%) is defined as how the percentage of the total liver is increasing in the early postoperative period. No correlation was found between ICG R15 and the liver regeneration rate in any of the three groups (not in the living liver donor group or the two groups of colorectal cancer patients with liver metastases who had or had not undergone chemotherapy). Some patients showed a relatively favorable hepatic reserve despite low ICG R15 levels and unfavorable liver regeneration rates, and some patients had high ICG R15 levels and liver regeneration rates that were virtually the same as in other patients, despite an unfavorable hepatic reserve.

**Figure 3:**
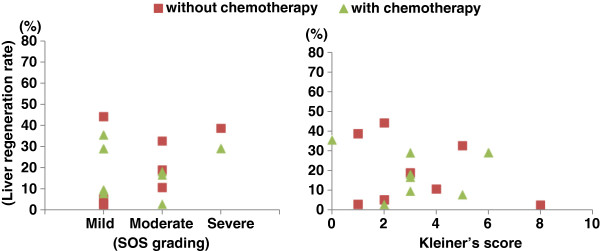
The relationships between the histopathological analyses and liver regeneration.

The results of the histopathological analyses of liver regeneration in colorectal cancer patients with liver metastases in the two groups (with and without preoperative chemotherapy) are shown (Figure 3). No correlation between SOS grading and the liver regeneration rates was found. Likewise, no correlation between Kleiner’s score and the liver regeneration rates was found. Some patients were assigned severe SOS grades, yet they showed a liver regeneration rate that was equal to or better than that in other patients. In some patients with a high Kleiner’s score (5 points or more), even those with chemotherapy-associated steatohepatitis (CASH), the liver regenerated more readily than in other patients.

## Discussion

A number of reports have indicated that conversion chemotherapy in colorectal cancer patients with multiple liver metastases allows the disease to be downstaged, thus rendering the disease resectable in such patients, and when combined with resection, contributing to improved survival. On the other hand, preoperative chemotherapy has also been previously reported to result in hepatic disorders such as steatohepatitis and sinusoidal dilatation. Such liver disorders may compromise postoperative liver regeneration, but other reports have suggested that surgical outcomes or complications are not adversely affected [19-25]. To analyze the effect on liver regeneration of chemotherapy-induced liver disorders, the postoperative rate of increase in liver volume in living liver donors (normal livers) and the postoperative rate of increase in liver volume for colon cancer patients with liver metastases in the chemotherapy and nonchemotherapy groups were investigated on the basis of volume in this study.

ICG R15 plays an important role in assessing hepatic reserve or determining the extent of hepatic resection volume in hepatocellular carcinoma. This study investigated whether, as a measure of hepatic reserve following preoperative chemotherapy, this ICG R15 or the severity of histopathological disorders that have been reported thus far can or cannot predict the rate of increase in liver volume.

**Figure 4:**
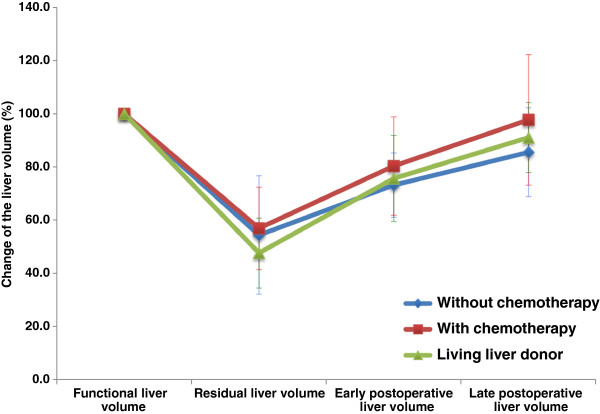
The results for liver regeneration versus the functional liver volume among the three groups.

This study, which is characterized by a small number of patients and a lack of serious postoperative complications in any of the groups, was a retrospective analysis, and the conclusions are by no means definitive. In addition, in colon cancer patients with multiple liver metastases, a residual liver volume of at least 35% must be ensured, and hepatic resection cannot be performed any sooner than 4 weeks after chemotherapy [26]. Hepatectomy was performed anatomically, using the hepatic vein as the landmark for dissection, where the surgeon made use of CUSA and the first assistant made use of a coagulation hemostatic device, with the intention of achieving the least possible congestion in the residual liver. Donor resection was by hepatectomy with uninterrupted blood inflow, whereas the Pringle method was used in approximately 40% of the colorectal cancer patients with liver metastases. Among the three groups, no significant differences in postoperative liver function (such as protein synthesis capacity or nutritional state) were noted in either the early or late postoperative periods (Table 4). The postoperative rate of increase in liver volume was about the same in the three groups, as was the rate of increase relative to resected volume. Even though living liver donor groups have advantageous conditions of liver generation such as younger age and no Pringle maneuver, the level of liver regeneration was the same in normal livers, tumor-bearing livers, and post-chemotherapy tumor-bearing livers, as estimated by liver volume [27]. To exclude the effects of the preoperative tumor loads, we have withdrawn the volume of the liver metastases from the total liver volume to calculate a “functional liver volume.” The results for liver regeneration versus the “functional liver volume” at the early postoperative period and late period are shown as almost even not only in the tumor-bearing two groups but also in the three groups involving the living liver donor groups (Figure 4). ICG R15 levels and histopathology, on the other hand, were variable, complicating the prediction of liver enlargement rates based on those results.

**Table 4:** Postoperative liver function

**Laboratory data (mean ± SE)**	**Living liver donor (*n*** **= 5)**	**Without chemotherapy (*n*** **= 9)**	**With chemotherapy (*n*** **= 8)**	*p *value*
**T-Bill (mg/dl)**				
**Preoperative**	0.69 ± 0.05	0.77 ± 0.18	0.45 ± 0.04	0.03
**5 postoperative days**	1.64 ± 0.44	1.42 ± 0.27	1.28 ± 0.27	0.81
**Early period**	0.93 ± 0.20	0.80 ± 0.21	1.60 ± 1.02	0.61
**Late period**	0.79 ± 0.11	0.72 ± 0.08	0.74 ± 0.08	0.97
**PT-INR**				
**Preoperative**	0.97 ± 0.02	0.96 ± 0.02	0.97 ± 0.03	0.82
**5 postoperative days**	1.11 ± 0.03	1.12 ± 0.03	1.10 ± 0.04	0.81
**Early period**	1.07 ± 0.05	1.01 ± 0.03	1.05 ± 0.04	0.68
**Late period**	0.95 ± 0.01	0.94 ± 0.03	1.02 ± 0.03	0.19
**Plt (**^**×**^**10**^**3**^**μl)**				
**Preoperative**	25.7 ± 1.6	22.5 ± 1.3	25.3 ± 1.9	0.35
**5 postoperative days**	19.7 ± 1.4	16.8 ± 1.3	16.8 ± 2.0	0.4
**Early period**	25.7 ± 1.9	16.8 ± 1.3	26.6 ± 3.7	0.16
**Late period**	22.1 ± 0.9	20.2 ± 2.9	19.4 ± 2.2	0.1
**Alb (g/Dl)**				
**Preoperative**	4.3 ± 0.1	4.0 ± 0.2	3.9 ± 0.2	0.22
**5 postoperative days**	3.1 ± 0.1	3.1 ± 0.1	2.9 ± 0.1	0.55
**Early period**	3.6 ± 0.2	3.5 ± 0.2	3.5 ± 0.2	0.74
**Late period**	4.2 ± 0.1	3.9 ± 0.2	3.4 ± 0.3	0.003

*p* values* compare variables by the Kruskal-Wallis test among the three groups.

It has been reported that preoperative chemotherapy does not adversely affect surgical outcomes or surgically related complications. In addition, the results of this study were derived from the comparative analysis of postoperative liver regeneration in living liver donors (normal livers). Both sources suggest that preoperative chemotherapy is a safe and effective option, provided that adequate residual liver volume is ensured, that the dosing regimen is followed (including preoperative chemotherapy washout in patients scheduled for surgery), and that anatomical hepatic resection is performed in a way that ensures blood flow will be controlled in the residual liver.

## Conclusion

In conclusion, as estimated on the basis of liver volume, the level of liver regeneration was the same among normal livers, tumor-bearing livers, and post-chemotherapy tumor-bearing livers. Liver regeneration was not adversely affected by the extent to which sinusoidal dilatation or steatosis was induced in noncancerous tissue by chemotherapy in patients scheduled for surgery. Preoperative chemotherapy may therefore be an effective option worth considering.

## Consent

Written informed consent was obtained from the patient for publication of this report and any accompanying images.

## Competing interests

The authors declare that they have no competing interests.

## Authors’ contributions

DT is the main author of this article. DT, TT and HN conceived this study. TT, HN and GW supervised the manuscript writing. YH and NI contributed to the collection of clinical information and data analysis. DT, TT and YH performed the pathological examination. HN, TT, YH and GW reviewed the manuscript and revised it thoroughly. All authors have read and approved the final manuscript.
